# Molecular Mind Games: The Medicinal Action of Cyclodextrins in Neurodegenerative Diseases

**DOI:** 10.3390/biom13040666

**Published:** 2023-04-12

**Authors:** Susana Santos Braga

**Affiliations:** LAQV-REQUIMTE (Associated Laboratory for Green Chemistry), Department of Chemistry, University of Aveiro, 3810-193 Aveiro, Portugal; sbraga@ua.pt

**Keywords:** 2-hydroxypropyl-β-cyclodextrin, Niemann–Pick disease, Parkinson’s, Alzheimer’s, autophagosomes, lysosome, cholesterol

## Abstract

Cyclodextrins are often used as molecular carriers for small active ingredients in medicine. Recently, the intrinsic medicinal activity of some of these compounds has been under investigation, mainly related to their ability to interfere with cholesterol and, therefore, prevent and treat cholesterol-related diseases such as cardiovascular disease and neuronal diseases arising from altered cholesterol and lipid metabolism. One of the most promising compounds within the cyclodextrin family is 2-hydroxypropyl-β-cyclodextrin (HPβCD), owing to its superior biocompatibility profile. This work presents the most recent advances in the research and clinical use of HPβCD against Niemann–Pick disease, a congenital condition involving cholesterol accumulation inside lysosomes in brain cells, Alzheimer’s and Parkinson’s. HPβCD plays a complex role in each of these ailments, going beyond the mere sequestering of cholesterol molecules and involving an overall regulation of protein expression that helps restore the normal functioning of the organism.

## 1. Introduction

### 1.1. Overview of Cyclodextrins

**Figure 1:**
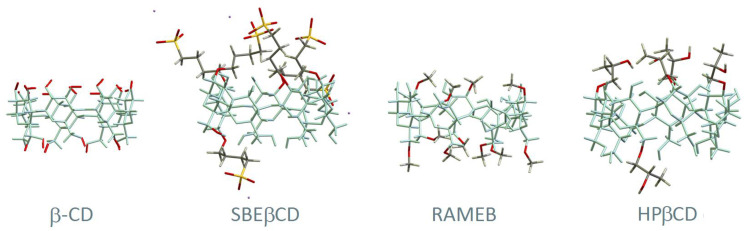
Schematic depiction of the structure of β-CD and its derivatives of pharmaceutical use. The hydroxyl groups located at the rims of β-CD, highlighted in red, serve as anchoring sites for chemical functionalisation. In the derivatives, only the functional groups (hydroxypropyl, sulphobutylether and methoxyl) are highlighted with colour. Adapted from Braga *et al.* [9] under a Creative Commons licence (CC BY 4.0).

Cyclodextrins (CDs) are cyclic oligosaccharides that can occur spontaneously in the natural environment by bacterial fermentation of starch [1,2] or be produced in a bioengineering facility by the action of enzymes on starch-rich substrates [3,4,5]. Naturally occurring cyclodextrins are usually named ‘native CDs’ and include α-CD, β-CD and γ-CD, having six, seven and eight glucose units, respectively. Their unique structure, first described in 1948 [6], features the D-glucose units linked by α-1,4-bonds, with the secondary and primary hydroxyl facing the upper and lower rims of the ring, and the inner cavity is lined with protons. The geometry of cyclodextrins makes them excellent solubilising agents for non polar molecules, with applications in controlled delivery, solubilisation and shelf-life extension of active ingredients in the pharmaceutical, cosmetic and food industries [7,8,9,10]. Chemical modification of cyclodextrins by placing functional groups at the available hydroxyl groups affords a variety of derivatives and allows fine-tuning the depth of their cavity and some physico-chemical properties such as solubility and aggregation. The structures of the cyclodextrin molecules that are most employed in pharmacy and medicine are depicted in Figure 1.

### 1.2. Cyclodextrin Regulatory Status and Pharmaceutical Use as Excipients

Native cyclodextrins are approved as excipients by the European Medicines Agency (EMA) [11] and they have the GRAS status, *i.e.,* ‘generally recognised as safe’ from the US Food and Drugs Administration (FDA) [12,13,14]. They are also approved as food additives by the Joint FAO/WHO Expert Committee on Food Additives (JECFA) [15,16,17]. Within chemically modified cyclodextrins, (2-hydroxy)propyl-β-CD (HPβCD) is one of the safest, being approved for parenteral, oral, rectal, dermal and ocular pharmaceutical formulations, while sulphobutylether-β-CD (SBE-β-CD) is used in parenteral and oral formulations. Methylated cyclodextrins have lower biological safety because of their higher lipophilicity and high potential to cause haemolysis. Because of this, most of the methylated derivatives of cyclodextrins are banned from pharmaceuticals. Nevertheless, one of these molecules is allowed for external use: randomly methylated β-CD (RAMEB), approved for topical administration, appears in the composition of nasal and ocular formulations [11].

In their classical pharmaceutical application, cyclodextrins are usually regarded as excipients with drug solubilising and stabilising action. Nowadays, one can find numerous examples of applications of cyclodextrins as excipients in commercial drugs. In liquid formulations, the most well-known are Sporanox® oral and injectable solutions that use HPβCD as solubiliser [18,19], with other examples being the use of SBEβCD in injectable antiviral remdesivir [20], *heptakis*-2,6-di-*O*-methyl-β-CD in chloramphenicol eye drops [21] and γ-CD in the paediatric form of the sedative midazolam [22]. Cyclodextrins are frequently found in solid dosage forms. An example of widespread use by female patients is Yaz®, a birth control and acne-treating tablet formulation containing the inclusion complex of 2βCD·ethinylestradiol [23]. Cyclodextrins also help formulate many other drugs into tablets, being found in association with antibiotics such as cefotiam, cefditorem and norfloxacin, anti-histamine drugs such as cetirizine and loratadine, the anti-hypertensive agent amlodipine, the expectorant bromhexin, anti-acids such as omeprazole and famotidine, the painkiller paracetamol, and numerous anti-inflammatory drugs (nimesulide, rofecoxib, meloxicam, piroxicam, tiaprofenic acid and aceclofenac); for a comprehensive list of cyclodextrins in commercial drug formulations, the reader is referred to the reviews of Puskás *et al.* [24] and Klein and Zöller [25].

### 1.3. Cyclodextrins in Medicine

The potential role of cyclodextrins as medicinal compounds started to attract the attention of researchers in the end of the XX century. This change of paradigm was triggered by the development of polysulphated cyclodextrin derivatives as part of a program investigating drug candidates to treat HIV (human immunodeficiency virus). Among other substrates, native cyclodextrins were used as polysulphonation scaffolds, yielding dozens of sulphated derivatives [26] of which some were able to stop HIV replication *in vitro*, but ultimately failed *in vivo* because HIV was able to become resistant to them [27]. In the early 2000’s, cholesterol was identified a target for HIV therapy [28], re-sparkling the interest in the medicinal use of cyclodextrins and broadening the span of research to a number of viral infections and cholesterol-associated metabolic disfunctions. Native and chemically modified cyclodextrins are able to bind to cholesterol with varying degrees of affinity. Those with a higher sequestering ability are β-CD and RAMEB, followed by HPβCD and then by TRIMEB, as determined *in vitro* in human vascular endothelial cells [29]. The high biocompatibility of HPβCD makes it typically the cyclodextrin of choice for medicinal applications that require cholesterol sequestering.

Currently, there is only one cyclodextrin approved as a medicine—this molecule, a *per*-6-sulphanylpropanoate derivative of γ-CD, is specifically designed to bind to the anaesthetic agent rocuronium bromide and revert its effect, helping patients recover their senses quickly (1.5 min) in postoperative settings [30,31]. Nevertheless, research in medicinal uses of cyclodextrins for a variety of diseases has grown exponentially in the last two decades [27] and these compounds are already in the drug development pipeline of several companies.

A relevant example is the field of cardiovascular disease. HPβCD has promising anti-atherosclerotic properties, helping to reduce inflammation in atherosclerotic tissue [32], dissolving cholesterol deposits in mouse arterial walls to facilitate its removal and reprogramming macrophage cells to metabolise cholesterol and form soluble oxysterol metabolites [33]. New research is revealing cardioprotective properties for HPβCD, particularly in preventing abdominal aortic aneurism; a study on a mouse model demonstrated its effectiveness in stopping disease progression [34].

A second example is the treatment of a genetic disease of the kidney characterised by abnormal build-up of cholesterol and lipids in the glomeruli (the cells responsible for filtration). This disease is called focal segmental glomerulosclerosis and it manifests by the presence of proteins in the urine (leaked by the lipid-damaged cells). HPβCD can restore normal renal function by removing the excess cholesterol from the glomeruli [35]. The use of HPβCD on human patients for the treatment of focal segmental glomerulosclerosis in phase IIa trials has been approved by the FDA [36]; the trials are currently being designed and they are set to start soon [37].

The neuroprotective role and cholesterol-regulating action of several cyclodextrins on cortical neuronal cultures is reported [38], with consequent changes in the activity of various proteins such as receptors, transporters and ion channels, and a significant role in the pathogenesis of several ischemic brain conditions, such as stroke [39], as well as in the progression of Alzheimer’s and Parkinson’s diseases. These two age-related neurodegenerative disorders currently have no effective therapeutic approaches to stop their progression. Therefore, the development of new drugs against them is the target of intense research. Impaired cholesterol trafficking and metabolism is a common trait in Parkinson’s and Alzheimer’s. It is also the root cause of a congenital brain disease, Niemann–Pick type C. This review describes the developments on the medicinal use of HPβCD as a cholesterol-regulating molecule in the field of neurodegenerative diseases, focusing on the three abovementioned conditions—the ones for which stronger scientific developments are available.

## 2. Hydroxypropyl-β-cyclodextrin in the Treatment of Niemann–Pick Disease

Niemann–Pick disease (NPD) of the type C is a congenital brain disease involving the excessive accumulation of cholesterol sphingolipids inside the lysosomes of brain cells. The disease has a genetic origin, being caused by malfunction in the gene encoding NPC1, a transport protein responsible for removing lipids out of the cells. Patients carrying the active mutation suffer from strong neurological dysfunction that manifests through symptoms such as ataxia, tremors, loss of muscle tone and even possible loss of vision. NPD starts to manifest during childhood and it causes progressive loss of bodily control and function that may ultimately lead to death [40].

### 2.1. Pre-Clinical Evaluation in Different Animal Models and Investigations into the Possible Mode of Action at the Cellular Level

Research on HPβCD as a possible drug for NPD was triggered by the lack of medicines for the disease and by the known ability of this cyclodextrin to sequester cholesterol and other lipids. Pre-clinical studies in mice using intravenous administration showed no results, thus leading to the conclusion that HPβCD cannot cross the blood–brain barrier [41]. Based on this conclusion, the authors further stated that the possible medicinal use of HPβCD in the treatment of NPD would require invasive intrathecal administration, that is, direct injection into the spinal canal. Using this route, it was possible to observe a beneficial action of HPβCD in mice [42] and cats [43] with NPD, which translated as slower progress of neural damage and reduced swelling in some regions of the brain. Murine studies further confirmed the ability of intrathecal HPβCD to delay the onset of clinical signs, reduce neurodegeneration by lowering cholesterol accumulation in neurons and significantly increase the lifespan of the subjects. Murines treated with HPβCD showed normal levels of autophagy and neuroinflammation biomarkers [44].

**Figure 2:**
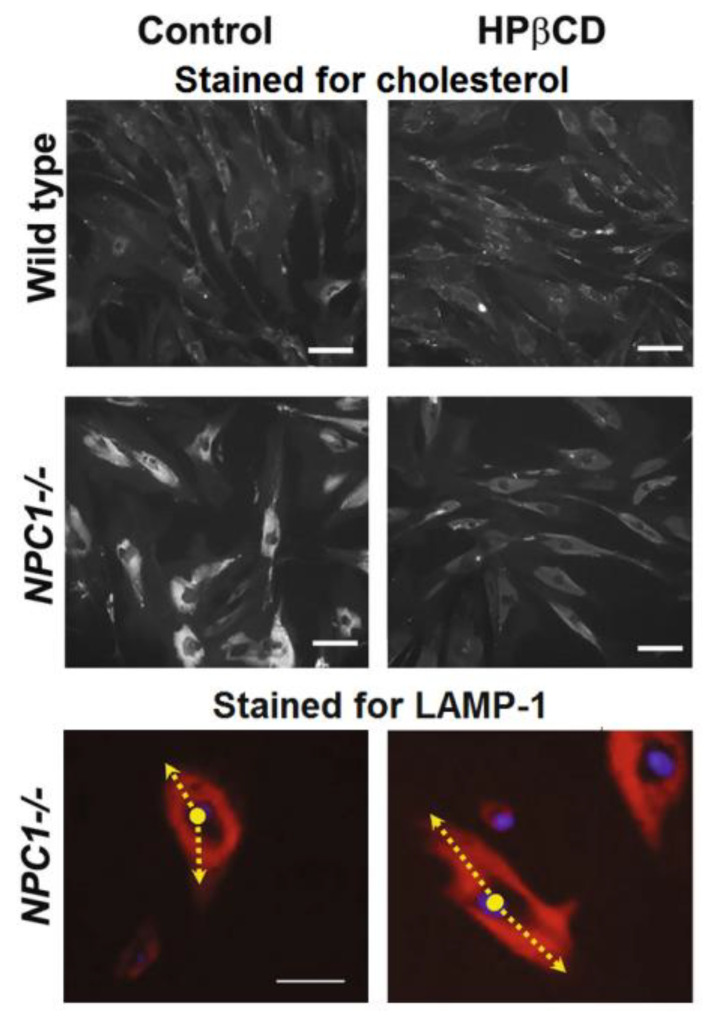
Immunostaining micrographs of healthy fibroblasts (wild type) and fibroblasts from a Niemann–Pick patient (*NPC1^−/−^*); scale bar = 50 μm. Top and middle rows show the distribution of cholesterol (in white, stained with filipin). Bottom row shows LAMP-1 distribution (in red, a lysosome marker) along with the cell nuclei (in blue). The arrow represents the distribution of LAMP-1 from the centre of the nuclei, depicting that the LAMP-1 protein is mostly confined to the area near the nuclear envelope in control *NPC1^−/−^* cells and distributed more widely throughout the cytoplasm when cells were treated with HPβCD. Cells were incubated with 1 mm HPβCD for 72 h prior to image collection. Images are a representative of at least three random fields of three experimental replicates. Adapted from Singhal *et al.* [45] under a Creative Commons licence (CC BY 4.0).

Investigation into the mode of action of HPβCD was carried out *in vitro* using fibroblasts from both healthy and Niemann–Pick-bearing donors. Results showed that HPβCD caused a clear reduction in the amount of intracellular cholesterol in the fibroblasts that carry the disease, making them appear, in micrographs, similar to the healthy control fibroblasts (Figure 2). The study further investigated the possible mechanisms of action of HPβCD to show that its cholesterol-lowering activity was mediated through the stimulation of lysosome-associated membrane protein 1 (LAMP-1), one of the proteins responsible for carrying lipids out of the cells [45].

**Figure 3:**
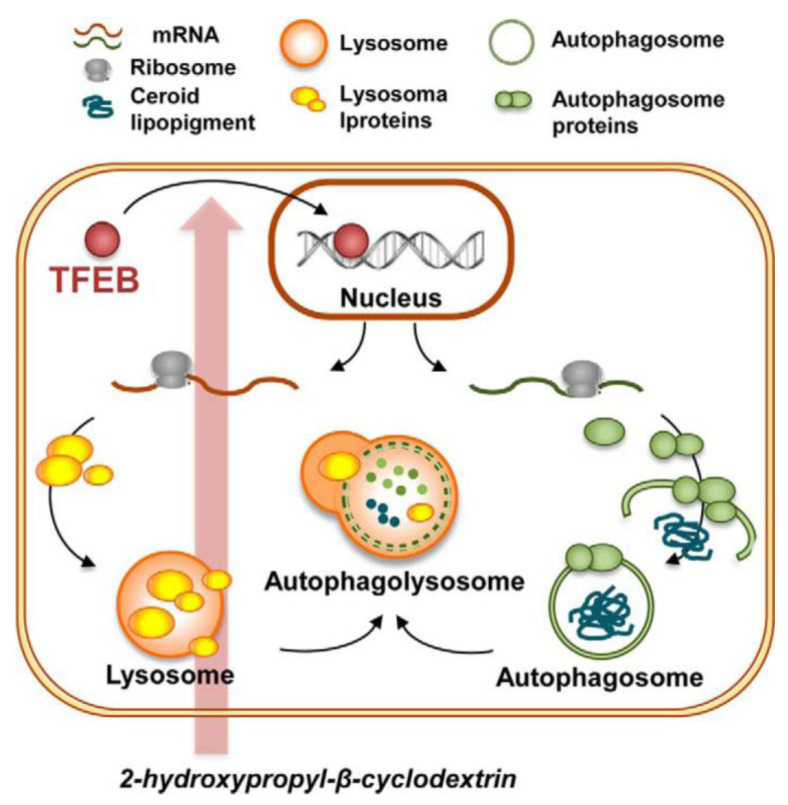
Schematic representation of the proposed adaptive cellular response to HPβCD treatment. HPβCD administration increases expression of the transcription factor EB (TFEB) in the cytoplasmatic ribosomes. Upon translocation from the cytoplasm to the nucleus, TFEB regulates the expression of genes involved in biogenesis and fusion of lysosomes and autophagosomes. As a result, HPβCD administration results in enhanced clearance of the autophagic substrate ceroid lipopigment in the cells of patients with congenital deficient elimination of this substance. Reproduced from Sarkar *et al.* [47] under a Creative Commons licence (CC-BY-NC-ND 3.0).

In another *in vitro* study with primary human fibroblasts, HPβCD was shown to upregulate cell autophagy, a process that is vital to cell maintenance as it helps eliminate unneeded proteins, macromolecules and proteolipid aggregates. The cellular bodies involved in this process, called autophagosomes, are linked to lysosomal activity, which means that their correct functioning is important for the lipid-eliminating action of lysosomes. Cellular uptake of HPβCD and accumulation into late endosomes and lysosomes resulted in activation of the cellular lipid clearance response mediated by the autophagy pathway in cells that have a defective lysosomal system (Figure 3) [46]. It is important to note that the fibroblasts used in this study were not obtained from NPD patients but rather from patients with another cell metabolism disorder (named neuronal ceroid lipofuscinosis). Nevertheless, the mode of action is transversal and applicable to NPD type C because it has been demonstrated that NPD patients also have compromised autophagy [47].

### 2.2. Clinical Trials and Compassionate Medicinal Use

The encouraging results obtained in pre-clinical trials and the lack of alternatives prompted the approval of HPβCD as an orphan drug for the compassionate treatment of NPD type C by both the FDA (in 2010) and the EMA (in 2013) [48]. Since then, its effect following intrathecal administration has been evaluated by several clinical trials. In one of these, patients were babies under 18 months of age and they showed restoration of neuronal cholesterol homoeostasis and a reduction of central nervous system pathology [49]. A trial enrolling 3 children with ages between 30 to 36 months showed that HPβCD improved cognitive scores and motor abilities, including the ability to swallow and to maintain balance; note that no placebo group was included [50]. A trial administering HPβCD intrathecally for a period of 3 years and enrolling 14 participants aged between 4 and 23 years also showed slowing of the progression of the disease [51].

In a larger trial enrolling 56 patients and having a double blind, 2:1 design, that is, 38 patients having HPβCD intrathecally (900 mg every 2 weeks for a total of 52 weeks) and 18 patients receiving a sham control, results found no statistically significant differences between HPβCD and the control [52,53]. Moreover, the trial showed non-neglectable incidence of hearing loss as a secondary effect of the treatment. HPβCD had been previously shown to have toxicity in outer hair cells of the ear [54]. Ultimately, the results of the trial caused HPβCD to fail clinical approval by the FDA as a commercial drug for Niemann–Pick Disease type C [55]. The small number of patients available for the trial and the unequal randomisation method it employed could be behind the poor results [56]. Further trials are under design to continue developing a clinical label for HPβCD (under the tradename adrabetadex) in NPD treatment [57].

## 3. Hydroxypropyl-β-cyclodextrin in the Management of Alzheimer’s Disease

Alzheimer’s disease is a neurodegenerative condition characterised by the accumulation of β-amyloid peptides in the brain [58,59]. Recent studies show that impaired cholesterol metabolism may contribute to increased risk of developing Alzheimer’s, because high cholesterol levels seem to correlate with higher amounts of internalisation of β-amyloid peptides into the neurons; in addition, cholesterol can interfere negatively with several proteases responsible for β-amyloid peptide degradation in the brain [60].

### 3.1. In Vitro and In Vivo Studies on Rodents Using Subcutaneous Administration

One of the main genes responsible for brain dyslipidaemia in Alzheimer’s disease is *APOE* [61]. This gene can be found in a variety of cells throughout the human organism, and are involved in the transport of cell lipids and cholesterol transport. *APOE4* polymorphism alters its normal functioning and causes accumulation of unsaturated triglycerides and lipid droplets in oligodendrocytes and reduced brain cell myelination from the early development stages of the patient [62].

The use of HPβCD as a possible therapy for patients with the *APOE4* polymorphism was recently evaluated in a mouse model. In vivo studies used mice that were genetically modified to carry the human *APOE^4/4^* gene; for eight weeks, mice were subcutaneously injected with either HPβCD (dose 2 mg/kg body weight twice weekly, *n* = 4) or with saline solution. Results showed significant reduction of cholesterol droplets in the hippocampus brain region of the treated group in regard to the control group. TEM-based ultrastructural analysis of *corpus callosum* of treated mice revealed that HPβCD increased the number of myelinated axons and led to thicker myelin sheaths. Cognition tests further showed improved cognition and memory skills, but not motor ability, in treated mice [62]. These results, while extremely promising, still lack an adequate mechanistic elucidation. The authors of the study evaluated the role of HPβCD on *APOE^4/4^* oligodendroglia cultures *in vitro*, having observed significant reduction of intracellular neutral lipid droplets (composed of cholesterol and neutral lipids such as triacylglycerides) [62]. However, direct action on brain tissue is most unlikely to be achieved from subcutaneous injection of HPβCD, because of its quasi-inability to cross the blod–brain barrier [41,63]. The action of HPβCD must, thus, be associated with effects on tissue other than the brain. It is known that peripheral APOE peptide isoforms, separated from those in the brain by the blood–brain barrier, are reported to be the main cause of development of Alzheimer’s symptoms [64]. Given that HPβCD will circulate freely throughout the body when administered by the subcutaneous route, peripheral tissue poses as a plausible target of action.

**Figure 4:**
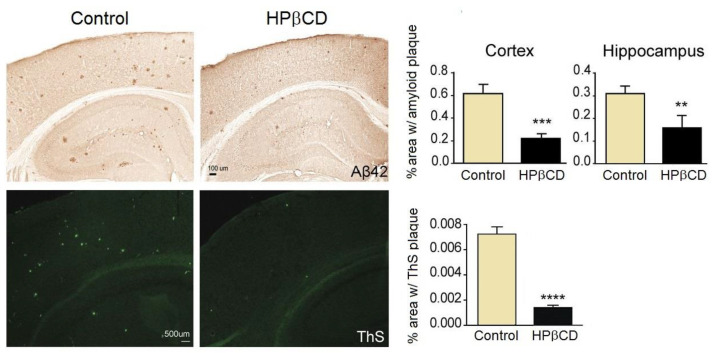
HPβCD treatment significantly reduces amyloid plaque burden in Tg19959 mice at four months of age. Top row: representative images of amyloid plaques detected with an Aβ42 antibody, along with the calculated percentage of area covered by amyloid plaques in cerebral cortex and hippocampus of Tg19959 mice treated with saline (control) or HPβCD. Bottom row: representative images of amyloid plaques stained with thioflavin (ThS), a fluorescent stain that binds to beta-sheet rich regions of amyloid plate. The chart depicts the percentage of brain tissue area covered by ThS-positive plaques. **, *p* < 0.01; ***, *p* < 0.001; ****, *p* < 0.0001; *n* = 9–10 in each group. All results were obtained and analysed from two separate animal studies. Reproduced from Yao *et al.* [65] under a Creative Commons licence (CC BY-NC-SA 3.0).

The beneficial role of HPβCD on the evolution of Alzheimer’s was demonstrated by the team of Yao *et al.* in a study with transgenic mice carrying the human gene for the amyloid peptide precursor (Tg19959 mice) [65]. These mice are thus designed to have congenital Alzheimer’s. They received treatment with HPβCD at a twice-weekly dose of 4 mg/kg body weight, starting 1 week after birth and ending when they were 4 months old. Mice under treatment showed better learning skills than the control group, but no improvements in motor skills. Mice were sacrificed after four months. Post-mortem brain histochemical studies showed reduced neuroinflammation and less β-amyloid peptide build-up (Figure 4), which seemed to be associated with a reduced amyloidogenic processing of the amyloid peptide precursor.

Yao *et al.* further observed that the reduction in β-amyloid plaques was accompanied by improvements in spatial learning and memory in Tg19959 mice [66].

Another *in vivo* study employed mice with several mutations, expressing a chimerical mouse-human amyloid precursor protein with the familial Alzheimer Swedish mutation (APP695swe), mutant presenilin 1 (PSEN1-dE9, a transmembrane protein that participates in the cleavage of β-amyloid peptides), and a dominant-positive, truncated and active form of sterol regulatory element binding factor 2 (SREBF2/SREBP2, to induce high levels of cholesterol in the brain and let it disrupt autophagic vesicles) [67]. The mice received a subcutaneous dose of 4 g/kg body weight of HPβCD every day for a period of 10 weeks. The treatment resulted in changes in levels and membrane distribution of key autophagosome and endosome-lysosome fusion proteins, which restored their ability to fuse and to perform autophagic action. Therefore, the clearance of β-amyloid peptide was improved. To better understand the role of HPβCD on the mouse Swedish mutation, *in vitro* studies were conducted [66]. To model the disease, mouse neuroblasts were modified to over-express Swedish mutant APP (SwN2), which caused them to have elevated cholesterol levels in the membrane. Upon treatment with HPβCD, cells showed reduced levels of membrane cholesterol, dramatically lower levels of β-amyloid peptide 42 and improved mitochondrial oxygen consumption.

These results served to demonstrate that HPβCD does have the ability to act directly on brain cells. For this to happen, administration needs to be conducted by a route that ensures HPβCD can reach brain tissue in adequate concentrations. Moreover, an adequate brain administration route can open the way for a possible action on the β-amyloid peptide aggregates located in the brain, because the ability of HPβCD to bind to fragments of the β-amyloid peptide has already been demonstrated by NMR and circular dichroism studies [68,69,70].

### 3.2. Exploring Intranasal Administration as a Brain-Targeting Route for HPβCD

Intranasal administration has been recently proposed as a suitable delivery route to bypass the blood-brain barrier and target the central nervous system, having as an advantage the fact that it is quite less invasive than intrathecal administration. While its efficacy in the administration of small molecules such as sedatives, anxiolytics and corticosteroids is well demonstrated [71], the delivery of substances of high molecular weight can be trickier, prompting a case-by-case evaluation. A study comparing the distribution and tissue concentrations of HPβCD in mice following administration by the intrathecal and the intranasal routes showed that in both cases it was taken up by all regions of the central nervous system, albeit the intranasal administration afforded lower levels than those obtained with the intrathecal one [72].

Evaluation of the activity of HPβCD, given by intranasal administration on a rodent model of Alzheimer’s, was reported [73]. Rats were administered with β-amyloid peptide 42 by direct injection to the hippocampus region of the brain and treated with HPβCD, which was administered to the nasal cavity in the form of mucoadhesive microspheres. While the hippocampus of rats that received no treatment showed signs of oxidative stress and apoptosis due to the increased levels of lipid peroxidation and reactive oxygen species, the hippocampus of rats that were treated with HPβCD showed significantly reduced levels of oxidative stress. These results presented some evidence of a protective action of HPβCD against the toxicity induced by the β-amyloid peptide 42 in the rat hippocampus; nevertheless, the authors of this study also noted that the excipients of the microparticles (sodium alginate or chitosan) could be involved in the therapeutic effect [73]. Therefore, the isolated therapeutic action of HPβCD on brain tissue affected with Alzheimer’s remains to be confirmed by further studies.

### 3.3. Ongoing Clinical Trial on HPβCD against Alzheimer’s

In November 2022, Cyclo Therapeutics, Inc, in collaboration with various research institutes, launched the first clinical trial to evaluate the potential medicinal efficacy, toxicological safety and tolerability of HPβCD in the treatment of early-stage Alzheimer’s disease [74]. The study, presently underway, enrols 90 human patients, aged 50 to 80 years, and is designed as a randomised, placebo-controlled, double-blind, parallel-group study. Two different test groups of thirty patients each will receive treatment, and the third group will receive a placebo. HPβCD will be administered by intravenous infusion over at least 4 h at doses of either 500 mg/kg or 1000 mg/kg every 4 weeks, for a total period of 6 months. Patients will be evaluated at the third and sixth month regarding memory, cognition, language, orientation and social skills. Results, if positive, will provide a strong contribution towards bringing HPβCD several steps closer to clinical application in the treatment of this neurodegenerative disease.

## 4. Expanding the Activity Range of HPβCD to Parkinson’s Therapy: Early Steps

Parkinson’s disease is a neurodegenerative condition associated with loss of dopamine-producing neurons at a midbrain region called the *substantia nigra*. One of the main causes of neuron loss in Parkinson’s disease is thought to be mitochondrial dysfunction, usually due to genetic causes, with the accumulation of toxic aggregates that end up causing cell death [75]; the main toxic components in these aggregates are fragments of α-synuclein (α-syn), a functional protein that helps regulate neurotransmission in healthy cells [76]. Mitochondrial imbalance was recently shown to be accompanied by impaired autophagy [77] (a similarity with Alzheimer’s disease).

One of the first studies that looked into the role of HPβCD on Parkinson’s resorted to an immortalised human H4 neuroglioma cell line (HTB-148, ATCC) [78]. Immortalised cells are easier to cultivate than primary cultures and, being of human origin, they pose as a good model for the disease in human patients. The cells were transfected with α-syn-EmGFP to mimic the alterations occurring in brain tissue affected by Parkinson’s (note: GFP, or green fluorescent protein, was used as a microscopy fluorescent marker to help track α-syn). One of the main outcomes of this study was to demonstrate that HPβCD reduced α-syn aggregates inside transfected cells. A mechanistical investigation into the mode of action of HPβCD showed that it was able to induce the clearance of α-syn aggregates through upregulation of autophagy, in a process that involved, among other pathways, LAMP-2-mediated increased autophagosome formation [78]. Note that LAMP-2 is the ‘sister’ protein of LAMP-1, which allows inferring that the mode of action of HPβCD in Parkinson’s is somewhat parallel to the one reported for NPD.

**Figure 5:**
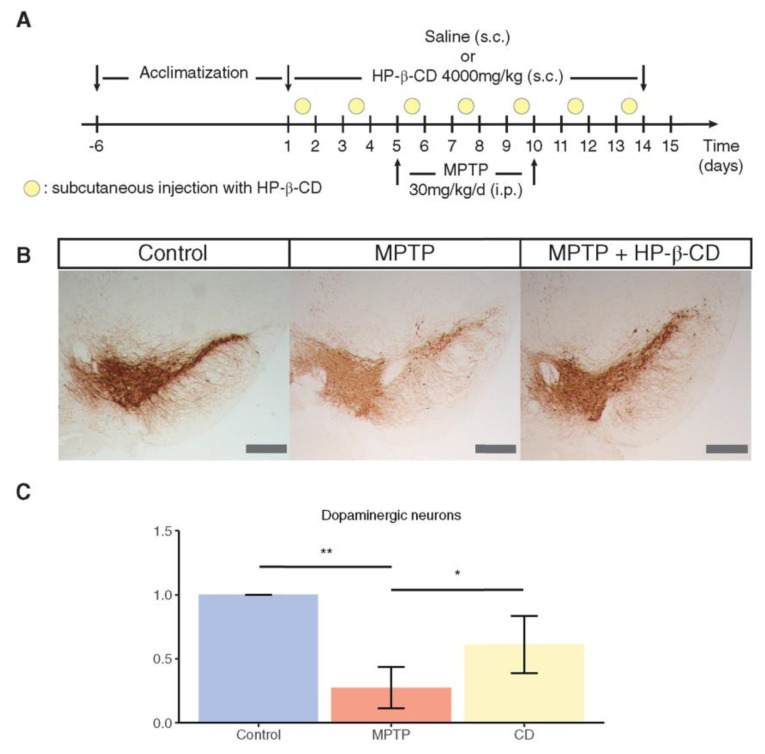
Treatment with HPβCD (represented as HP-β-CD in this figure) protects against toxicity of MPTP. (**A**) Treatment scheme for the generation of MPTP-induced subacute Parkinson’s disease mice model and subsequent treatment with HPβCD. (**B**) Representative mouse midbrain sections stained for tyrosine hydroxylase (TH) in control, MPTP or HPβCD-treated mice (bar = 400 μm). (**C**) Stereological quantification of the TH levels in mouse sections normalised to control levels. Statistical analysis was performed using Kruskal–Wallis and Dunn’s tests for selected comparisons. * *p* < 0.05, ** *p* < 0.01. Reproduced from Jarazo *et al.* [77] under a Creative Commons Licence (CC BY 4.0).

To mimic the complex and tridimensional structure of brain tissue, an *in vitro* study used tridimensional midbrain organoids as model for the Parkinson’s affected brain [77]. The organoid was generated from pluripotent cells with mutation in one of the main genes that cause the disease. Results from this model showed that treatment with HPβCD helped restore the growth of dopaminergic neurons. The next step of this study was to evaluate the preventive role of HPβCD *in vivo* using a mouse model of the disease. Test animals received HPβCD subcutaneously at a dose of 4 g/kg body weight twice a week for a period of two weeks; at the fifth day of the study, mice received a neurotoxin (MPTP, 1-methyl-4-phenyl-1,2,3,6-tetrahydropyridine) to induce Parkinson’s disease. Histological observation of mouse midbrain sections showed that HPβCD significantly reduced the loss of dopaminergic neurons in treated mice (Figure 5) [77].

A method for using HPβCD to reduce the death of dopaminergic neurons in Parkinson’s and related diseases was recently patented [79], thus demonstrating the interest of this compound for this class of diseases.

## 5. Overview and Future Outlook

The present review describes the application of HPβCD as a medicine for neurologic diseases. In this context, it is important to bear in mind that HPβCD does not currently have a medicinal label, and that its use in humans is restricted to the compassionate treatment of patients suffering from NPD. In the clinical trials conducted with patients suffering from this disease, HPβCD has afforded improvement of the disease symptoms, albeit with lack of statistical significance due to the low number of recruited patients. A few of these studies have further demonstrated that HPβCD contributes to extend of lifespan of these patients. These results are prompting continued studies with a broader test group which may ultimately lead to clinical approval.

The mode of action of HPβCD in NPD, initially thought to be related to its ability to sequester cholesterol and remove it from the cells with over accumulation of this lipid, is now known to be more complex. In vitro studies on cell cultures from NPD patients showed that the increase in the removal of intracellular cholesterol build-up involves stimulation of the expression of the protein LAMP-1, upregulation of autophagosome activity, and re-establishment of the normal lysosome activity.

Possible application of HPβCD in the treatment of two neurodegenerative diseases, Parkinson’s and Alzheimer’s, is under investigation. In Parkinson’s disease, *in vitro* results provide indications that the mode of action of HPβCD in helping reduce brain cell damage may parallel the one reported for NPD. Using Parkinson’s-mimicking neuroglioma H4 cells, it was shown that HPβCD activates a pathway that involved inducing translocation of LAMP-2 to target cell sites, which increases the formation of autophagosomes and restores the clearance mechanisms necessary to eliminate the toxic aggregates of α-syn that are the root cause of neural damage.

The targets of action of HPβCD in Alzheimer’s are trickier to clarify because of the different administration routes tested in different studies, which result in a radical difference in the bioavailability of the compound in brain tissue. In rodent models of Alzheimer’s, HPβCD was administered subcutaneously, and was practically unavailable in brain tissue because of its neglectable permeation of the blood-brain barrier. A possible explanation for the therapeutic action of HPβCD when given subcutaneously is that it may contribute to regulate the peripheral expression of peptides that act as a background cause of the disease, slowing its development without acting directly on the brain. On the other hand, a study in which HPβCD mucoadhesive microparticles were administered to rats by the intranasal route to bypass the blood-brain barrier showed a direct protective effect against oxidative stress induced by exposure to β-amyloid peptide 42. While the number of available studies is low and more studies are needed to help clarify the mode of action of HPβCD on Alzheimer’s, all reports are coherent in showing promising results on halting or, at least, containing neurodegeneration. Following these positive outcomes, the first clinical trials on the use of HPβCD on human patients with early-stage Alzheimer’s has recently been launched. The study is currently underway, and for this reason no results have yet been released. The relevance of this study is, nevertheless, undeniable—besides serving to demonstrate the usefulness of HPβCD in the target pathology, any positive outcomes, particularly regarding safety, will pave the way for application in other neurodegenerative diseases, namely Parkinson’s, for which studies on humans are unavailable.
